# Association between the left-sided atrial septal pouch and the cryptogenic stroke – an updated systematic review and meta-analysis

**DOI:** 10.1038/s41598-025-21285-y

**Published:** 2025-10-27

**Authors:** Kamil Tyrak, Jakub Batko, Jakub Hołda, Kacper Jaśkiewicz, Mateusz Hołda

**Affiliations:** 1 HEART, Heart Embryology and Anatomy Research Team, Department of Anatomy, Jagiellonian University Medical College, Cracow, Poland; 2 Doctoral School in Medical Sciences and Health Sciences, Jagiellonian Universtiy Medical College, Krakow, Poland; 3 Department of Cardiac Surgery and Transplantology, National Medical Institute of the Ministry of Interior and Administration, Warsaw, Poland; 4 Division of Cardiovascular Sciences, The University of Manchester, Manchester, UK; 5 HEART - Heart Embryology and Anatomy Research Team, Department of Anatomy, Jagiellonian University Medical College, Kopernika 12, Kraków, 31-034 Poland

**Keywords:** Cryptogenic stroke, Left-sided septal pouch, Left atrium, Interatrial septum, Anatomy, Cardiology

## Abstract

Cryptogenic stroke, named embolic stroke of undetermined source, refers to patients for whom the etiology of embolism remains unknown. Recently a new cardiac entity, the left-sided septal pouch (LSSP), has been identified as a possible source of thromboembolic events. In the current study, we aimed to perform a systematic review of the literature on this topic and to determine the association between the LSSP presence and occurrence of cryptogenic stroke using meta-analytical methodologies. A detailed search of electronic databases for studies that compared the presence of LSSP in subjects with cryptogenic stroke and non-stroke controls was performed. Data were extracted and pooled into a meta-analysis. We included eight studies in the meta-analysis, in which there were a total of 506 patients with cryptogenic stroke and 1600 patients in the control group. The pooled prevalence of LSSP among cryptogenic stroke patients was 31.6% (95% CI: 20.6–43.8). In the non-stroke control group the pooled prevalence of LSSP was 22.0% (95% CI: 15.0-29.8). The meta-analysis showed that there is a higher risk of cryptogenic stroke in patients with LSSP than in patients without LSSP (OR: 1.57, 95% CI: 1.23–2.01; *p* < 0.01). A subgroup meta-analysis of transesophageal echocardiography studies demonstrated the same association (OR: 1.59; 95% CI: 1.21–2.08; *p* < 0.01). In studies where the mean patient age was < 60 years, LSSP was associated with a higher risk of cryptogenic stroke (OR: 1.67; 95% CI: 1.22–2.29; *p* < 0.01). However, in studies where the mean patient age was > 60 years, the association was not statistically significant (OR: 1.58; 95% CI: 0.75–3.32).

**Supplementary Information:**

The online version contains supplementary material available at 10.1038/s41598-025-21285-y.

## Introduction

Cryptogenic strokes account for around 40% of all ischemic strokes and their origin remains unknown despite standard clinical workup^1^. Some of cryptogenic strokes may be associated with cardiac interatrial septum, especially atrial septal variants, such as: patent foramen ovale and atrial septal aneurysm^2–5^. Recently the new cardiac entity, the left-sided atrial septal pouch (LSSP), have been identified as a possible source of thromboembolic events^6,7^. The left-sided atrial septal pouch is an anatomical variant of the interatrial septum, formed as a result of an incomplete fusion of the patent foramen channel components in postnatal life^6^. This kangaroo-like pouch communicates exclusively with the left atrium^8^. The morphology of LSSP may predispose to blood stasis and thus thrombus formation^6]– [7^.

Many direct evidences of formation of thrombotic material within LSSP and its direct involvement in the development of ischemic stroke have been delivered in clinical case reports^9–16^. The main mechanisms contributing to thrombogenesis in LSSP include blood stasis and local flow disturbances (the pouch’s dead-end nature promotes blood pooling and low-velocity flow, creating an environment prone to thrombus formation), endothelial dysfunction and local inflammation (which lead to an increase in prothrombotic factors), and an association with atrial fibrillation (studies have suggested that LSSP is linked to a higher incidence of atrial fibrillation, a well-established risk factor for cardioembolic stroke)^6,7,17,18^. Conditions such as heart failure, mitral stenosis, or left atrial hypertension can worsen blood stagnation in the pouch. Moreover, reduced laminar flow from the right-sided pulmonary veins might further decrease washout of the LSSP, facilitating thrombus formation^19^.

The identification of LSSP as a potential risk factor for cryptogenic stroke carries important clinical implications. Given the anatomical predisposition of LSSP to thrombus formation, its presence may serve as a marker for embolic risk, particularly in patients with unexplained strokes. Current stroke prevention strategies primarily focus on well-established cardiac sources, such as patent foramen ovale or atrial fibrillation, yet the role of LSSP remains underappreciated in clinical guidelines. In previous years, few original studies focused on the association between the LSSP and stroke development were published, with the results mostly being inconclusive. A meta-analysis published in 2018 showed that there is a significant association between presence of the LSSP and cryptogenic stroke^20^. Since the meta-analysis was published, 11 more studies that tried to determine the role of the LSSP as a risk factor of cryptogenic stroke were published^3,7,10,21–28^. Therefore, in this study we sought to perform updated systematic review and meta-analysis focused on the association between LSSP and embolic stroke of undetermined source. A better understanding of LSSP-related stroke mechanisms could lead to improved risk stratification, personalized antithrombotic therapy, and the potential development of targeted interventions, including transcatheter approaches for LSSP elimination. By providing updated meta-analytical evidence on the association between LSSP and cryptogenic stroke, this study may contribute to shaping future recommendations for stroke prevention and guide clinicians in the diagnostic workup of embolic stroke of undetermined source.

## Materials and methods

### Search strategy

The Preferred Reporting Items for Systematic Reviews and Meta-Analyses (PRISMA) guidelines for systematic reviews and meta-analyses were used^29^. A systematic literature search of the PubMed, EMBASE, Scopus, Web of Science and Science Direct databases were performed. All publications published before November 1, 2024 were analyzed. The terms “septal pouch” OR “atrial septal pouch” AND “stroke” were used to search the electronic databases. The search was not restricted by language or time of publication.

### Eligibility assessment

We considered studies in which the interatrial septum morphology was assessed in humans, where the LSSP variant was described in patients with cryptogenic stroke, and comparison to the control group of patients without stroke was performed. Studies such as case reports, editorials, conference abstracts, and duplicate reports from the same study were excluded. Two independent investigators screened titles and abstracts of found records to identify eligible publications. Full texts of chosen records were also independently screened and reviewed by the same two investigators. Potential discrepancies regarding the inclusion of studies were resolved by discussion and consensus among all investigators.

### Data extraction

Two independent investigators independently extracted data from the included studies. The extracted data included the year, country, study design, sample size, characteristics of each group (stroke vs. non-stroke) and number of LSSPs identified in each group. Disagreements regarding the data extraction were resolved by discussion and consensus among all investigators.

### Statistical analysis

Statistical analysis was performed using the IBM SPSS Statistics v. 28.0.1.0 (IBM Corporation, Armonk NY, USA; 2021. Available from: https://www.ibm.com/products/spss-statistics) and MetaXL v. 5.3 from (EpiGear International Pty Ltd, Wilston, Queensland, Australia; 2016. Available from: http://epigear.com/index_files/metaxl.html) to calculate the pooled prevalence of LSSPs. The Risk Of Bias In Non-randomized Studies of exposure effects (ROBINS-E) tool was used to evaluate the bias of included studies^30^. LFK index test was performed to analyze the symmetry of funnel plots in assessing the reporting bias^31^. We assessed heterogeneity by using Cochran’s Q test and calculating I^2^ values. Results were interpreted according to guidelines in the Cochrane Handbook, Chap. 10.10.2^32^. I^2^ values for heterogeneity assessment were categorized as follows: < 30% considered as low, between 30 and 75% considered moderate, and > 75% considered high. P-values < 0.05 were considered statistically significant. The meta-analysis was performed using the random-effects model.

## Results

**Fig. 1:**
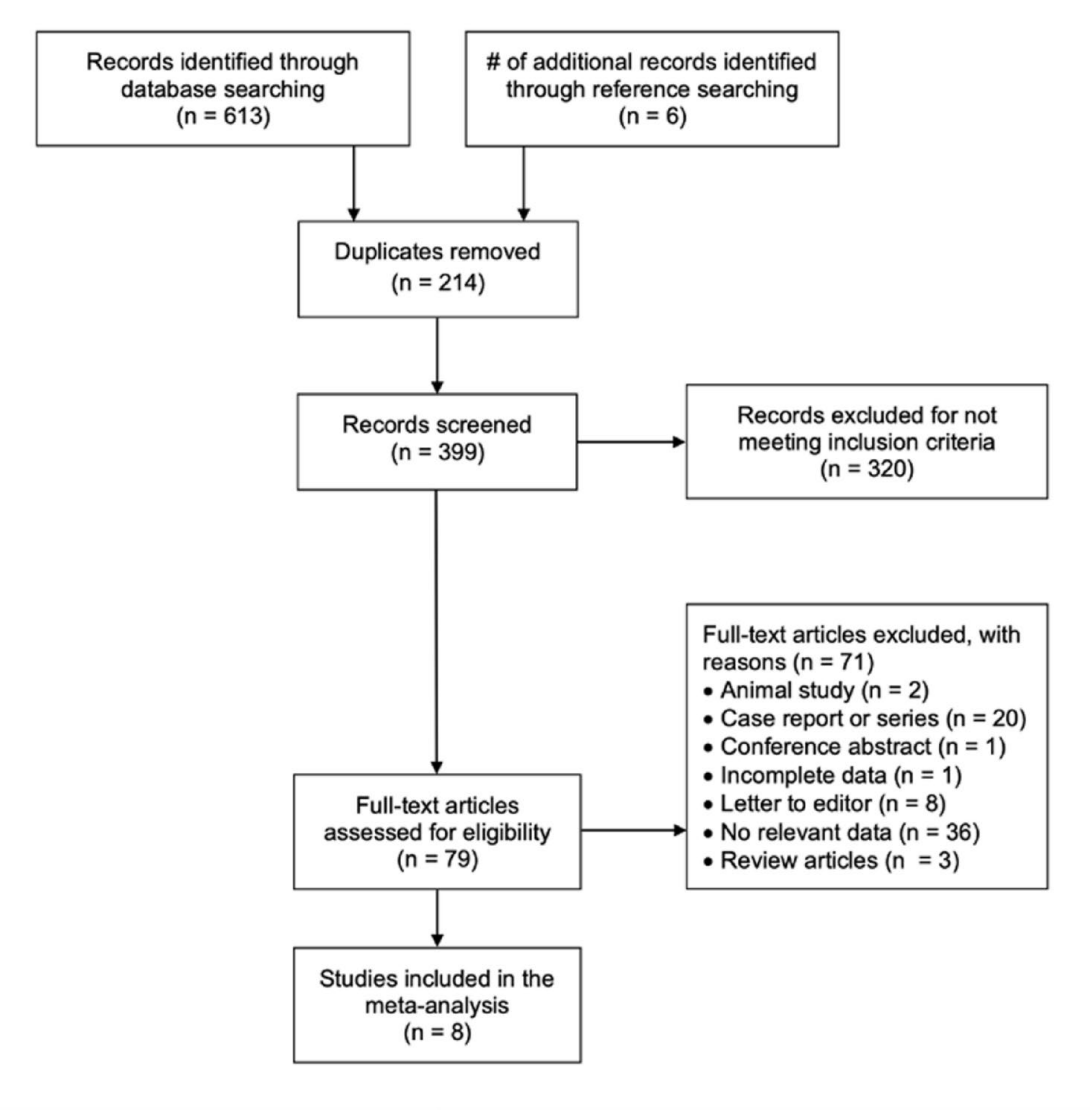
Flow chart demonstrating a selection of studies for meta-analysis. n, the number of records.

A systematic search identified 633 studies and six additional studies were identified by manual reference search, of which 410 remained after exclusion of duplicates (Fig. 1). A total of 321 studies were excluded for not meeting inclusion criteria. Remaining 89 studies were assessed by full text for potential eligibility. In the next step studies were excluded for being case reports, conference abstracts, editorials, reviews, book chapters, or dual publications or because they reported irrelevant data that were not related to septal pouches in the heart. Four publications were morphological studies^6,8,33,34^, six publications were imaging studies and did not refer clinical correlations with strokes^17,35–39^, one study was rejected due to lack of comparison with the control group^28^ and one study rejected because in both the control and stroke groups all patients had atrial fibrillation and were eligible for ablation (this publication was also characterized by significant heterogeneity of the control and stroke groups)^27^. In summary, eight publications that met our criteria were finally included in the meta-analysis^3,7,16,21–25^.

**Fig. 2:**
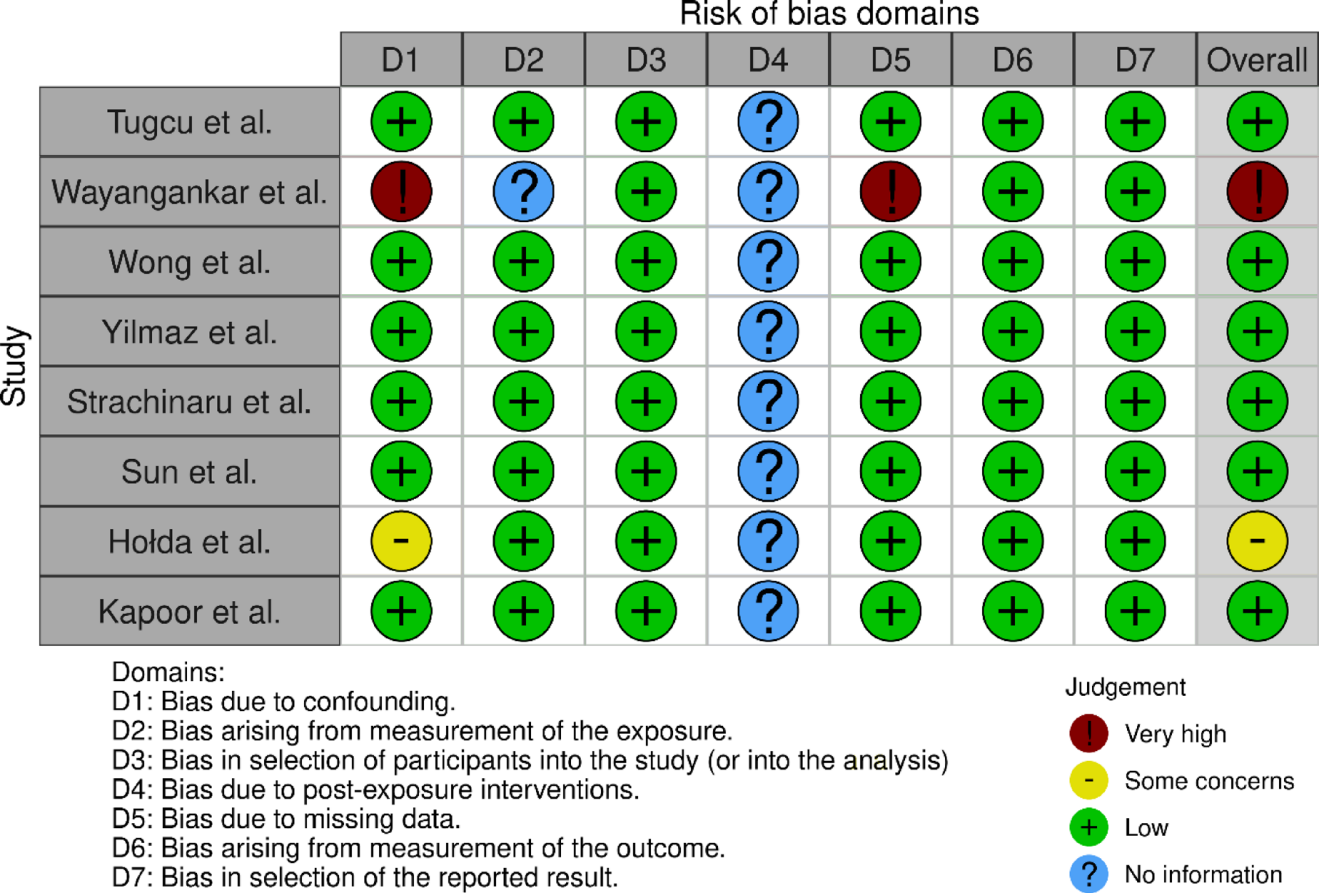
The risk of bias assessment using the ROBINS-E (Risk Of Bias In Non-randomized Studies of Interventions of exposure effects) tool.

Next, we assessed each of the included studies for the assessment of patients with cryptogenic stroke, as well as the association between cryptogenic stroke and the occurrence of LSSP. Seven included papers reported a relationship between the occurrence of cryptogenic stroke and the presence of LSSP. During data collection for the previous meta-analysis (2018)^20^, we contacted Sun et al.^25^ to clarify whether the strokes described in their work were cryptogenic strokes. The authors confirmed that all strokes described in their publication were cryptogenic strokes of unknown etiology^25^. The characteristics and results of the eight studies included in the meta-analysis are presented in Table 1. The majority of studies were transesophageal echocardiography studies (seven out of eight)^3,7,16,21,23–25^, and in one study the imaging method was cardiac computed tomography^22^. All included publications describe cases of adult patients, however, there is a significant difference between the studies as shown in Table 1. Due to the limited number of studies included in the analysis, bias assessment was performed using the LFK method (an index between − 1 and + 1 signifies no detectable bias)^31^. The Doi plot and LFK index results showed no significant asymmetry (LFK index = -0.91) (Supplementary Figs. 1 and 2), indicating a low risk of bias. In the risk of bias assessment using the ROBINS-E tool (Fig. 2), the study by Wayangankar et al. was found to have a very high risk of bias due to confounding and missing data biases^21^. The study by Hołda et al. was classified as having a moderate risk of bias due to concerns related to confounding bias^7^. The remaining studies included in the analysis were assessed as having a low risk of bias.

**Table 1:** Characteristics of studies included into meta-analysis.

Study	Year	Country	Imaging method	Type of study	Preregistration	Interatrial septum related exclusion criteria	Cryptogenic stroke patients						Non-stroke patients (control)				
							All patients, *n*	Patients with LSSP, *n*	Patients without LSSP, *n*	Age, years, mean ± SD	Group characteristic	Cryptogenic stroke definition	All patients, *n*	Patients with LSSP, *n*	Patients without LSSP, *n*	Age, years, mean ± SD	Group characteristic
Tugcu et al.^3^	2010	USA	TEE	Case-control study	Local ethics committee	Presence of PFO, closed pouch, right-sided septal pouch and ASD	69	22	47	69.7 ± 8.4	Consecutive patients with first acute ischemic stroke	Modified TOAST criteria (modification not specified)	156	46	111	67.0 ± 8.4	NOMAS database patients: age, sex, and race/ethnicity matched
Wayangankar et al.^21^	2013	USA	TEE	No information	-*	Presence of PFO and ASD	66	9	57	-*	Consecutive patients undergoing TEE	Ischemic stroke with absence of atrial septal defect or shunt, atrial fibrillation, > 4 mm aortic arch plaque, and flow-limiting carotid disease (as determined by ultrasound or computed tomographic imaging)	500	51	449	-*	Consecutive patients undergoing TEE
Wong et al.^16^	2015	USA	TEE	Retrospective study	Local ethics committee	Presence of PFO and ASD	23	7	16	Mean: 57 Range: 16–90^#^	Consecutive patients undergoing TEE	Modified TOAST criteria: ischemic strokes with multiple competing identified etiologies were excluded from the cryptogenic category	106	15	91	Mean: 57 Range: 16–86^#^	Consecutive patients undergoing TEE
Yilmaz et al.^22^	2016	Turkey	CT	Case-control study	Local ethics committee	Presence of PFO, ASD and ASA	40	13	27	42.0 ± 6.7	18–55 years old patients with the acute stroke	Modified TOAST criteria: stroke of cause that was unknown despite extensive routine diagnostic examinations	40	10	30	42.5 ± 7.1	Sex-matched healthy controls
Strachinaru et al.^24^	2016	Belgium	TEE	Retrospective study	Local ethics committee	-*	45	7	38	55.0 ± 13.0	Consecutive patients admitted into the stroke unit undergoing TEE	The patients who had no definite source of cardioembolism, large artery atherosclerosis, or small artery disease despite extensive vascular, cardiac, and serologic evaluation	223	42	181	65.0 ± 15.0	Consecutive patients undergoing TEE
Sun et al.^25^	2016	China	TEE	Retrospective study	Local ethics committee	Presence of PFO and ASD	31^†^	10	21	61.0 ± 12.0 (total population)	Consecutive patients undergoing TEE†	Ischemic stroke without definite source of thromboembolism	293	48	245	61.0 ± 12.0 (total population)	Consecutive patients undergoing TEE
Hołda et al.^7^	2018	Poland	TEE	Retrospective study	Local ethics committee	Presence of PFO, ASD and ASA	126	70	56	43.1 ± 11.1	Consecutive patients with first acute ischemic stroke	Modified TOAST criteria: cryptogenic stroke as an ischemic stroke in patients who had no definite source of cardioembolism, no large artery atherosclerosis, and no small-artery disease and for whom the cause of stroke was not defined despite extensive evaluation	137	56	81	45.3 ± 10.0	Consecutive patients, age and race/ethnicity matched
Kapoor et al.^23^	2021	USA	TEE	Retrospective study	Local ethics committee	Presence of PFO and ASD	67	29	38	58.2 ± 1.2	Consecutive patients undergoing TEE	Modified TOAST criteria: ischemic strokes with multiple competing identified etiologies were excluded from the cryptogenic category	144	41	103	55.9 ± 1.2	Consecutive patients undergoing TEE

* information not provided by the authors in the publication.

^#^ Information regarding the standard deviation was not provided; only mean with age range was reported.

† Information that all stroke patients were cryptogenic was obtained after contact with the first author.

ASA, atrial septal aneurysms; ASD, atrial septal defect; CT, cardiac multidetector computed tomography; LSSP, left-sided septal pouch; NOMAS, Northern Manhattan Study; PFO, patent foramen ovale; TEE, transesophaegal echocardiography. n, number of patients.

**Fig. 3:**
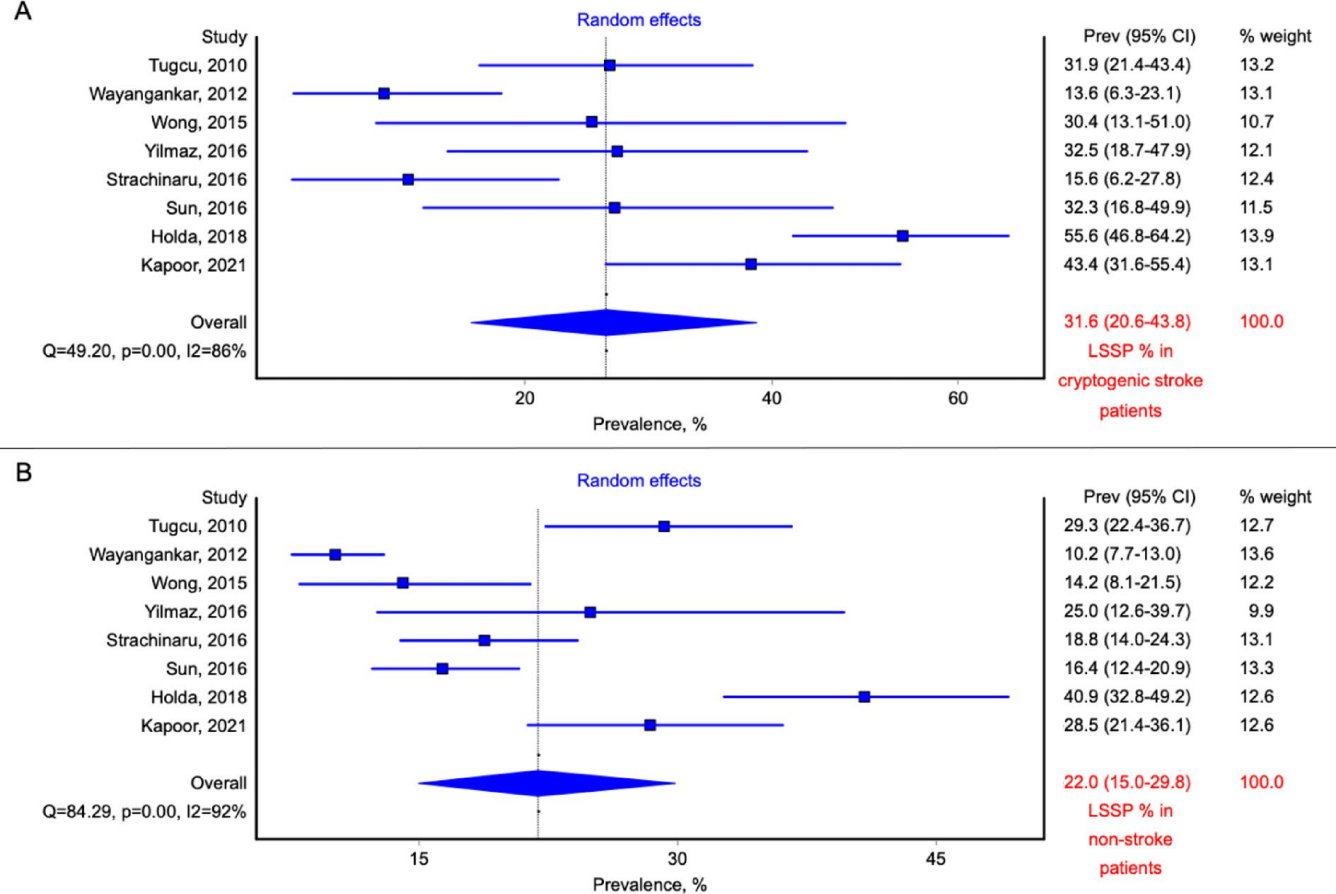
Forest plot of the prevalence of the left-sided septal pouch (LSSP) in (**A**) cryptogenic stroke patients and (**B**) non-stroke control patients. The studies are presented along the vertical axis and depicted as squares, the size of which is proportional to the calculated weight of each study. The overall effect estimate is positioned at the bottom and illustrated by a diamond.

**Fig. 4:**
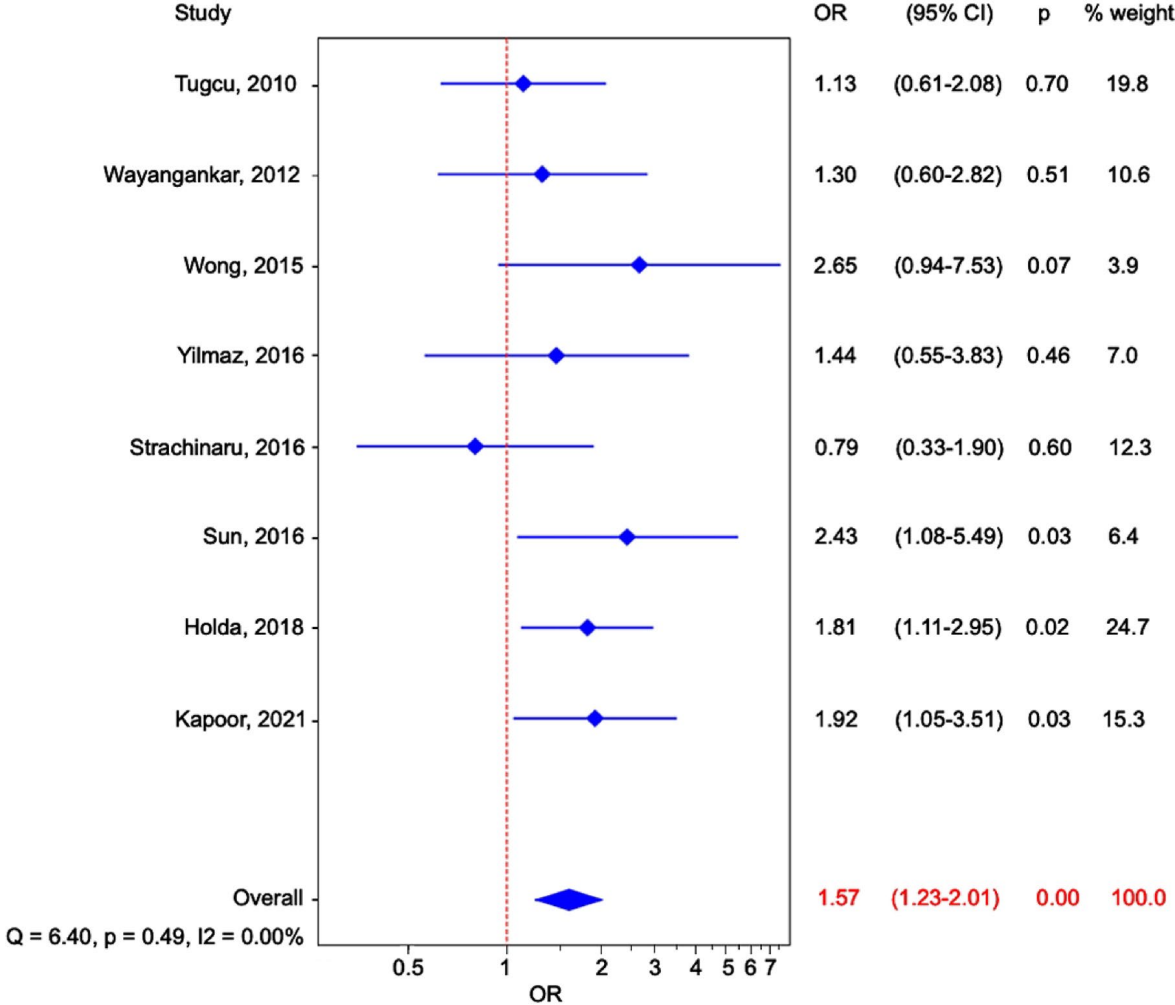
Forest plot representation of the results from the studies comparing cryptogenic stroke patients with non-stroke controls. The studies are presented along the vertical axis and depicted as squares, the size of which is proportional to the calculated weight of each study. The overall effect estimate is positioned at the bottom and illustrated by a diamond.

In total, 506 patients with cryptogenic stroke were recognized (mostly based on modified TOAST criteria, Table 1). Among patients with cryptogenic stroke, 167 cases of LSSP were identified. The pooled prevalence of LSSP among cryptogenic stroke group was 31.6% (95% CI: 20.6–43.8, random effects model; Fig. 3A). Control, non-stroke group consisted of 1600 patients, with 329 individuals with present LSSP. The calculated pooled prevalence of LSSP in non-stroke group was 22.0% (95% CI: 15.0-29.8, random effects model; Fig. 3B). The meta-analysis showed that the risk of cryptogenic stroke was higher in patients with present LSSP (OR: 1.57; 95% CI: 1.23–2.01; *p* < 0.01; Fig. 4). Calculated heterogeneity was low in the analysis (Q = 7.14, *p* = 0.41, I^2^ = 2%).

**Fig. 5:**
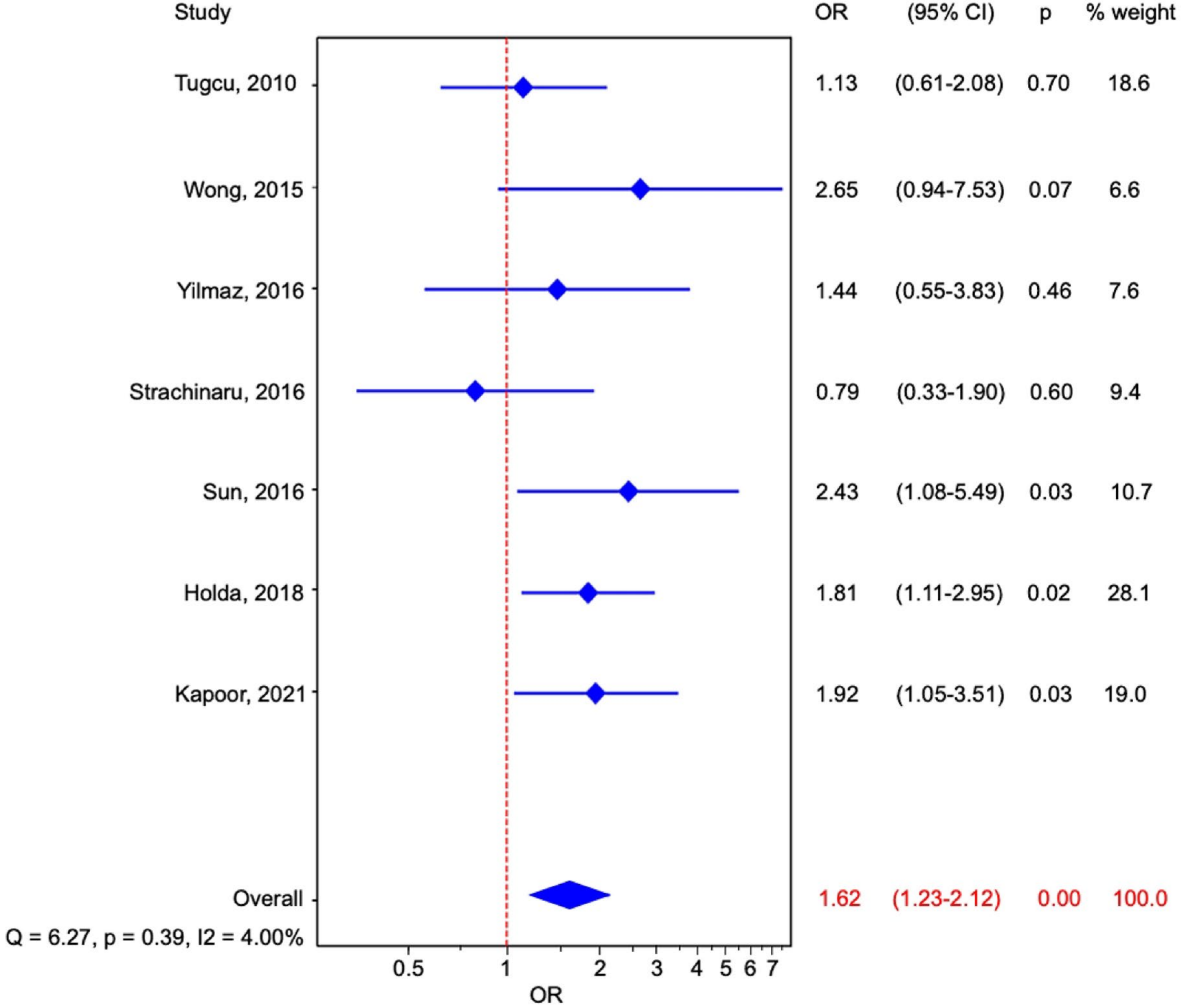
Forest plot representation of the results from the studies comparing cryptogenic stroke patients with non-stroke controls, excluding the study by Wayangankar et al. due to its high risk of bias. The studies are presented along the vertical axis and depicted as squares, the size of which is proportional to the calculated weight of each study. The overall effect estimate is positioned at the bottom and illustrated by a diamond.

**Fig. 6:**
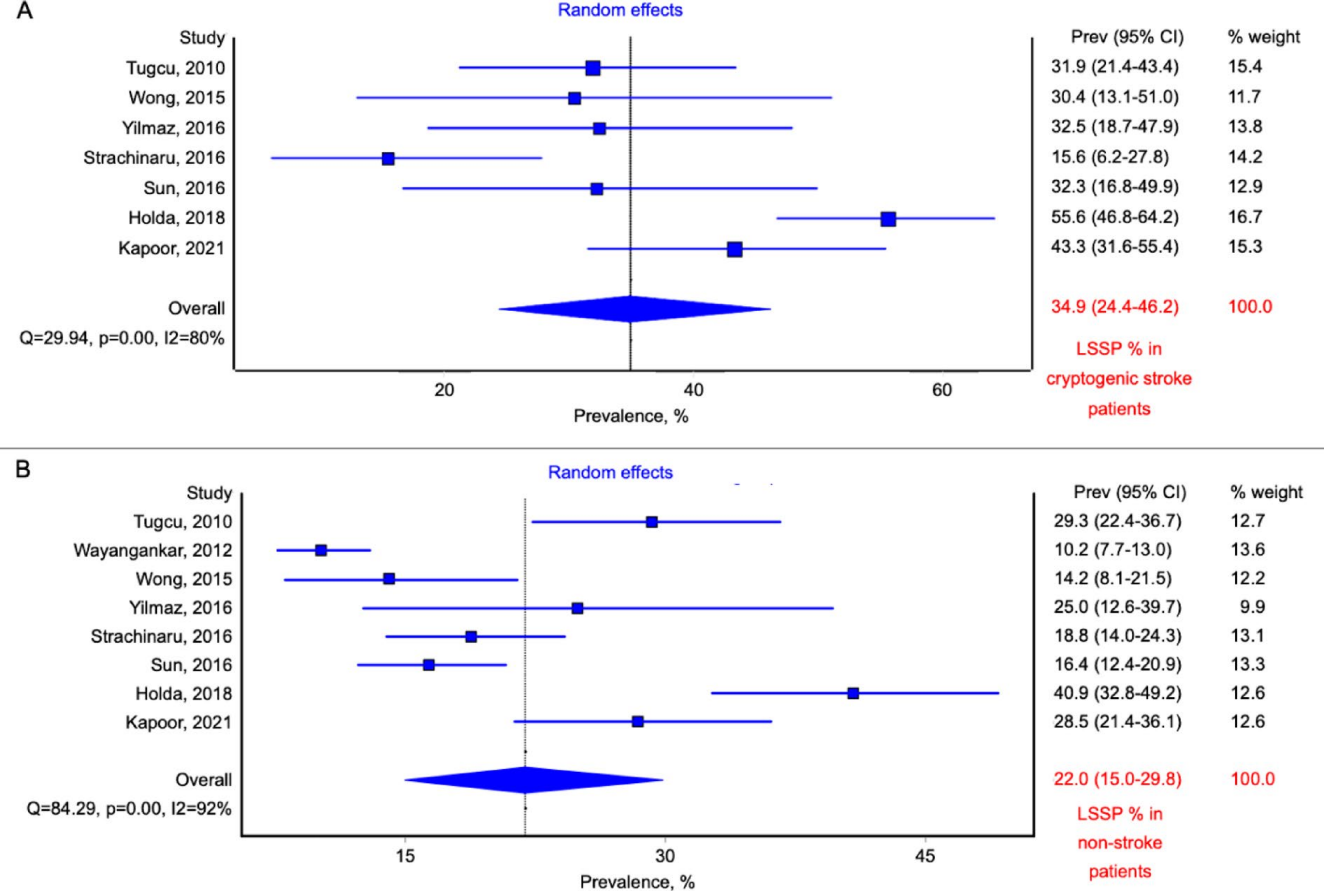
Forest plot of the prevalence of left-sided septal pouch (LSSP) in (**A**) cryptogenic stroke patients and (**B**) non-stroke control patients, excluding the study by Wayangankar et al. due to its high risk of bias. The studies are presented along the vertical axis and depicted as squares, the size of which is proportional to the calculated weight of each study. The overall effect estimate is positioned at the bottom and illustrated by a diamond.

An additional meta-analysis was conducted after excluding the study by Wayangankar et al.^21^ due to its high risk of bias. However, the findings remained similar to those of the meta-analysis that included this study (OR: 1.62; 95% CI: 1.23–2.12, *p* < 0.01; Q = 6.27, *p* = 0.39, I² = 4%; Fig. 5). The pooled prevalence of LSSP in the cryptogenic stroke group, after excluding the study by Wayangankar et al.^21^, was 34.9% (95% CI: 24.4–46.2, random-effects model; Fig. 6A), while in non-stroke group was 24.2% (95% CI: 17.6–31.5, random-effects model; Fig. 6B).

A further meta-analysis was performed after excluding the studies by Tugcu^3^, in which both the cryptogenic stroke and control groups were the oldest (70.6 ± 9.0 and 67.0 ± 8.4 years, respectively), and by Strachinaru^24^, which reported a statistically significant age difference between the study and control groups (55.0 ± 13.0 vs. 65.0 ± 15.0 years; *p* < 0.01). After excluding these two studies, the meta-analysis yielded results consistent with those of the analysis that included them, demonstrating that the risk of cryptogenic stroke remained higher in patients with LSSP than in those without LSSP (OR: 1.83; 95% CI: 1.38–2.45; *p* < 0.01; Q = 1.96, *p* = 0.88, I² = 0%; Supplementary Fig. 3).

The study by Yilmaz et al.^22^ was the only one utilizing computed tomography imaging, whereas all other studies employed transesophageal echocardiography for detecting LSSP. A subgroup meta-analysis of transesophageal echocardiography studies demonstrated that the risk of cryptogenic stroke was higher in patients with LSSP than in those without it (OR: 1.59; 95% CI: 1.21–2.08; *p* < 0.01; Supplementary Fig. 4).

An additional subgroup meta-analysis was performed based on the mean patient age, using a cutoff of 60 years. In the subgroup of studies where the mean patient age was younger than 60 years, the risk of cryptogenic stroke was higher in individuals with LSSP than in those without the pouch (OR: 1.67; 95% CI: 1.22–2.29; *p* < 0.01; Supplementary Fig. 5). In the subgroup of studies where the patients’ mean age was greater than 60 years, the OR was 1.58; however, this result was not statistically significant, as the confidence interval crossed the threshold of no effect (95% CI: 0.75–3.32; Supplementary Fig. 6).

## Discussion

The present, updated systematic review and meta-analysis confirmed a significant relation between the occurrence of LSSP and a cryptogenic stroke. The specific features of LSSP, such as its small size, dead end, orientation with apex directed downward and local inflammation within pouch may predispose to the formation of thrombi inside pouch^6^. The previous studies have also shown that the presence of LSSP may be associated with an increased risk of atrial fibrillation^17^. These factors may be a rational explanation why the occurrence of LSSP is associated with an increase in the risk of cryptogenic stroke. Additionally, the risk of thrombi formation within the LSSP may be exacerbated by any disturbances to the blood flow within the left atrium (brisk laminar blood flow from the right pulmonary veins is believed to be a protective mechanism) as well as conditions leading to peri-wall blood stasis (mitral stenosis, various atrial arrythmias, high ventricular pressure and heart failure)^6]– [7^.

Since the previous meta-analysis on this topic, published in 2018, which showed that cryptogenic stroke occurred 1.5-times more frequently in patients with an LSSP than in patients without the LSSP^20^, some new important studies have been published that could potentially change this conclusion^23,27,28,40^. Nevertheless, the passage of 5 years has not brought a breakthrough in this matter and our current study confirms that the presence of LSSP is associated with almost 1.5-times higher incidence of cryptogenic stroke compared to the control group. Our systematic review also identified a meta-analysis conducted by Amin et al. in 2023, which included a total of 10 retrospective, observational studies published between 2010 and 2022^41^. However, after reviewing the studies included in the Amin et al. meta-analysis, we concluded that the analysis was not performed correctly, as it included irrelevant studies (study by Kapoor et al.^23^, by Celik et al.^26^ and by Steyaert et al.^28^ and, therefore, does not adequately address the research question posed (see discussion of individual studies below).

Interestingly, over 5 years period only one new study was published that met inclusion criteria of current meta-analysis^23^. Study by Kapoor et al. investigated different types of strokes for the presence of LSSP and only in the case of cryptogenic strokes a statistically significant difference in the occurrence of LSSP compared to the control group was found (LSSP prevalence significantly higher in the cryptogenic subgroup compared with the non-cryptogenic subgroup, *p* = 0.02)^23^. Furthermore, study by Celik et al. showed that LSSP is associated with an increased risk of ischemic brain alterations (OR: 3.57, 95% CI: 0.51–2.09, *p* < 0.01), however, all cerebral ischemic strokes, not just cryptogenic ones, were considered in this study^26^. Also, the study by Goertz et al. have showed that the presence of LSSP is associated with 2.9-fold increased hazards of ischemic brain lesions (95%CI: 1.2–7.4, *p* = 0.024), but not just cryptogenic strokes were analyzed^40^. For these reasons, these two articles were not included in our analysis. Study by Steyaert et al. that retrospectively analyzed transesophageal echocardiograms in a large cohort of patients that had experienced ischemic strokes (148 had cryptogenic stroke and 978 had strokes of known origin) concluded in multivariate analysis that LSSP was independently associated with cryptogenic stroke (*p* = 0.019)^28^. However, because no comparison with the non-stroke controls was performed by Steyaert et al. this study was excluded from our meta-analysis. On the other hand Michałowska et al. showed no association between the occurrence of LSSP and incidence of stroke^27^. However, this publication may be an example of a poorly designed study, as all types of ischemic strokes were investigated (not only cryptogenic stroke). Moreover, all patients in both the stroke and control groups had atrial fibrillation, thus taking into account fact that atrial fibrillation is one of the main risk factors for stroke, this study was excluded from the meta-analysis.

The limitations of this meta-analysis are related to the limitations of each of the included studies. In each of the studies included in the analysis, the definition of cryptogenic stroke was based on the differently modified TOAST criteria, however, different group selection criteria in each of the studies may distort the final results (Table 1). One of the main limiting factor may be the age of the patients. The interatrial septum undergoes continuous remodeling throughout life. During the postnatal life the patent foramen ovale channel transforms into.

a septal pouch (young adults) and further into a smooth septum (elderly)^6^. Among the.

8 included publications, relatively young groups of patients were described in studies performed by Yilmaz et al.^22^, Wong et al.^16^, Hołda et al.^7^ and Kapoor et al.^23^ (Table 1). On the contrary in the study performed by Tugcu et al. both the cryptogenic stroke group and the control group were the oldest (70.6 ± 9.0 and 67.0 ± 8.4 years respectively)^3^. In addition, study by Strachinaru et al. contained groups with significantly different age (55.0 ± 13.0 vs. 65.0 ± 15.0-years-old; *p* < 0.01)^24^. Both publications by Tugcu et al.^3^ and Strachinaru et al.^24^ have a large weight (24.4% in total), which may affect the results of the meta-analysis and the actual impact of the presence of LSSP in individual age groups may be different. In the study by Wayangankar et al. information on the age of the patients was not included^21^.

Furthermore, our age-based subgroup analysis revealed a particularly strong association between LSSP and cryptogenic stroke among patients younger than 60 years. This finding indicates that younger individuals with LSSP may have an elevated risk of cryptogenic stroke, which could be attributable to age-specific factors such as variations in interatrial septal remodeling, differences in atrial flow dynamics, or increased thrombotic susceptibility at earlier stages of life^6,7^. The precise mechanisms underpinning this heightened vulnerability in younger patients remain to be fully elucidated. Conversely, in patients older than 60 years, the influence of LSSP on stroke risk is less clear. Our analysis did not demonstrate a definitive association within this older subgroup, suggesting the possibility that other age-related factors or comorbidities may overshadow the impact of LSSP. Given the uncertainty regarding the role of LSSP in the older population, additional targeted studies are warranted to explore and clarify this relationship more comprehensively. Further research focusing on age-specific differences in the pathophysiological implications of LSSP is necessary to enhance our understanding of its contribution to embolic stroke risk. Investigations should particularly address whether younger patients with LSSP would benefit from targeted screening programs or preventive interventions, potentially guiding clinical management strategies and reducing stroke incidence among susceptible younger individuals.

Other factors that may influence the results of the meta-analysis may also be identified. The ethnic background of evaluated patients may be considered, but so far no ethnic differences in the LSSP occurrence were showed and they should be accounted for in further studies. Moreover, the used cardiac imaging method may have influence on underdiagnosis of the LSSP in the patients form both stroke and non-stroke groups. Notably, the study by Yilmaz et al.^22^ as the only one utilizing computed tomography for LSSP detection, while all other studies relied on transesophageal echocardiography. A subgroup meta-analysis of transesophageal echocardiography-based studies confirmed a significant association between LSSP and cryptogenic stroke, reinforcing the validity of echocardiographic imaging in identifying this potential stroke risk factor. Although the transesophageal echocardiography with saline contrast administration should be preferred over the contrast enhanced cardiac computed tomography in the identification of LSSPs they both may be used in clinical practice to identify patients with the LSSP^39^. More research on LSSP imaging methods in the human heart should also be conducted to indicate the best method for LSSP identification. Finally, the associations between LSSP and other types of cerebrovascular (e.g. transient ischemic attack) and systemic thromboembolic events should also be investigated in further studies.

As clinical studies and meta-analyses have confirmed the association between the presence of a LSSP and cryptogenic stroke, there is a pressing need to consider the role of this structure in official clinical guidelines. Understanding the clinical implications of the LSSP could enhance risk stratification and inform management strategies, especially for patients with unexplained embolic events or atrial fibrillation, thereby contributing to more personalized approaches in cardiovascular care. A recent case report demonstrated that atrial septal defect occluder implantation may be a viable therapeutic option for eliminating the LSSP, confirming the technical feasibility of mitigating the pathological influence of this structure on the human body^42^. Recent clinical trial demonstrated the safety and efficacy of transcatheter exclusion of the LSSP in patients at high risk for cryptogenic stroke. Transcatheter LSSP elimination for secondary stroke prevention (clamping and compressing the pouch after transseptal puncture) may potentially pave the way for novel structural interventions targeting the interatrial septum^43^. Future research should focus on prospective, longitudinal studies to better define the causal relationship between LSSP and cryptogenic stroke. While retrospective studies and meta-analyses, including the present study, have demonstrated a significant association, prospective cohort studies could help determine whether LSSP is an independent risk factor for embolic events. Additionally, large randomized controlled trials evaluating the efficacy of targeted interventions, such as transcatheter LSSP closure, in reducing stroke risk could provide valuable insights into potential therapeutic approaches. Furthermore, future studies should investigate potential genetic and demographic predispositions to LSSP-related stroke, as well as explore the role of coexisting cardiovascular conditions, such as atrial fibrillation, in modifying the risk profile of affected patients. These research efforts will be critical in shaping future guidelines for stroke prevention and optimizing patient management strategies.

## Conclusions

Our meta-analysis confirms that there is statistically significant association between LSSP presence and occurrence of cryptogenic stroke. LSSP presence is associated with increased cryptogenic stroke risk.

## Supplementary Information

Below is the link to the electronic supplementary material.

Supplementary Material 1

Supplementary Material 2

Supplementary Material 3

Supplementary Material 4

Supplementary Material 5

Supplementary Material 6

Supplementary Material 7

## Data Availability

All data generated or analysed during this study are included in this article. Further enquiries can be directed to the corresponding author.
