# Supplementary material for: Association between the left-sided atrial septal pouch and the cryptogenic stroke – an updated systematic review and meta-analysis
Source: Sci Rep. 2025 Oct 27;15:37432. doi: 10.1038/s41598-025-21285-y (PMC12559741; doi:10.1038/s41598-025-21285-y)
Supplement: Supplementary file 1 — Supplementary Material 1 [file 41598_2025_21285_MOESM1_ESM.docx]

**Supplementary Figures Legends**

Supplementary Figure 1. Assessment of publication bias using DOI plot. LFK index results showed no significant asymmetry (LFK index = -0.91). LFK: Luis Furuya-Kanamori index; OR: odds ratio.

Supplementary Figure 2. Assessment of publication bias using funnel plot. OR, odds ratio; ES, effect size.

Supplementary Figure 3. Forest plot representation of the results from the studies comparing cryptogenic stroke patients with non-stroke controls, excluding the study by Tugcu et al. and Strachinaru et al.

Supplementary Figure 4. Forest plot representation of the results from the transesophageal echocardiography studies comparing cryptogenic stroke patients with non-stroke controls.

Supplementary Figure 5. Forest plot representation of the subgroup meta-analysis results from studies comparing cryptogenic stroke patients with non-stroke controls, focusing on studies with a mean patient age lower than 60 years.

Supplementary Figure 6. Forest plot representation of the subgroup meta-analysis results from studies comparing cryptogenic stroke patients with non-stroke controls, focusing on studies with a mean patient age greater than 60 years.

In Supplementary Figures 3-6, the studies are presented along the vertical axis and depicted as squares, the size of which is proportional to the calculated weight of each study. The overall effect estimate is positioned at the bottom and illustrated by a diamond.
