# Supplementary figures and images for: Association between the left-sided atrial septal pouch and the cryptogenic stroke – an updated systematic review and meta-analysis
Source: Sci Rep. 2025 Oct 27;15:37432. doi: 10.1038/s41598-025-21285-y (PMC12559741; doi:10.1038/s41598-025-21285-y)

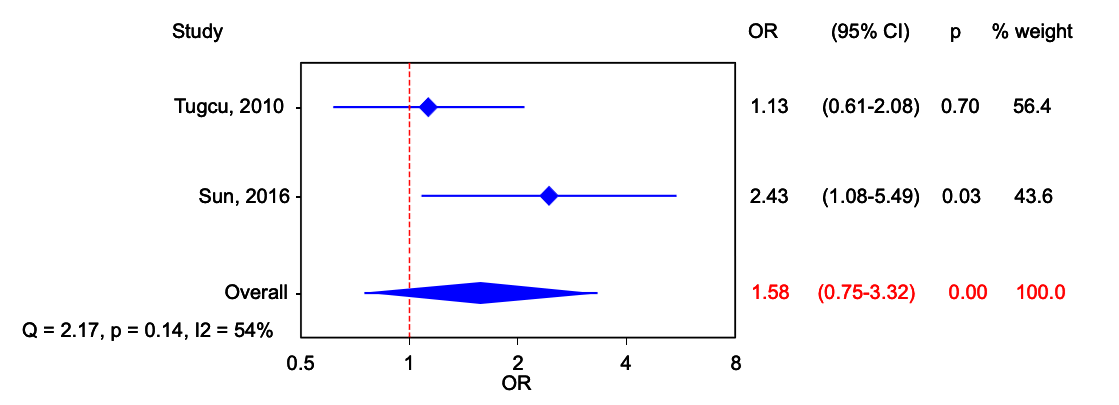

Supplement: Supplementary file 2 — Supplementary Material 2 [file 41598_2025_21285_MOESM2_ESM.png]

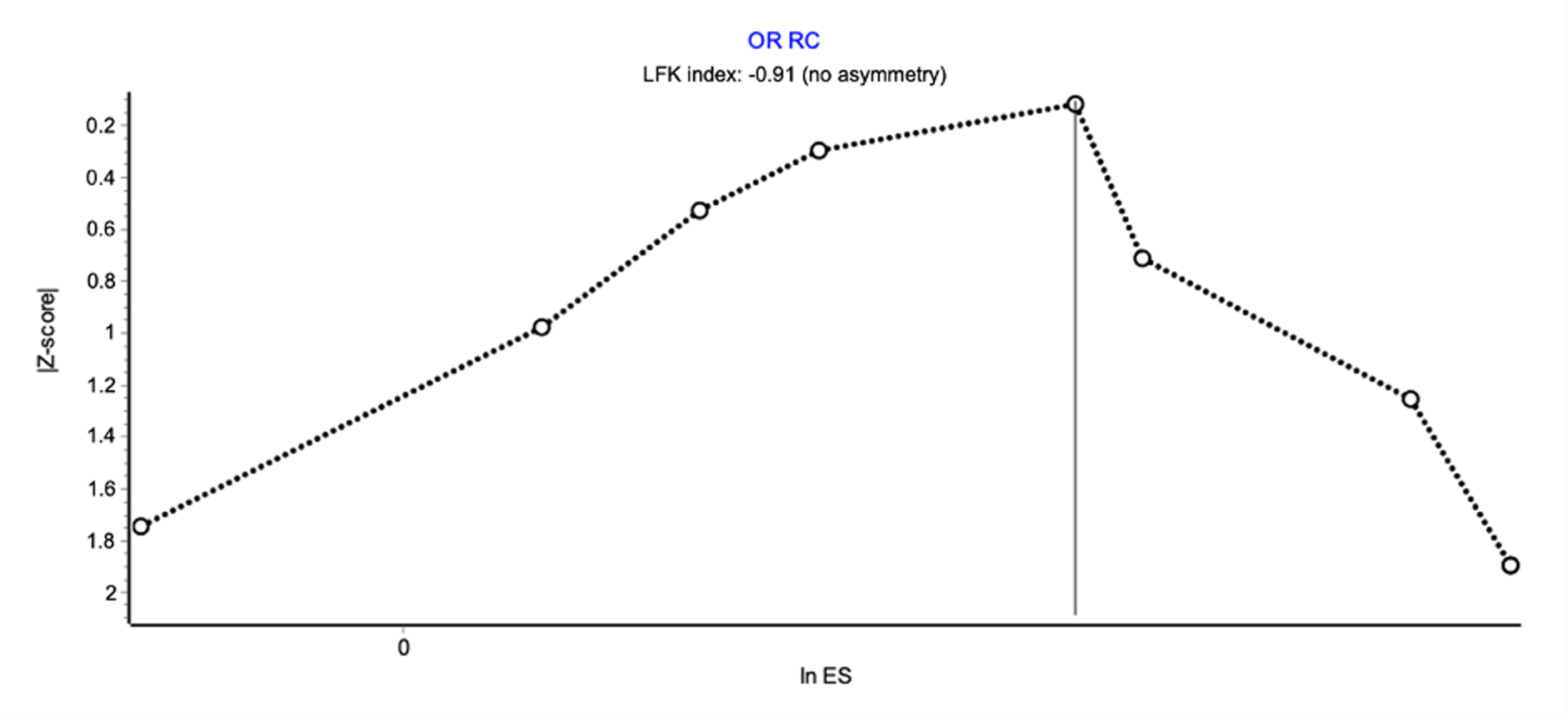

Supplement: Supplementary file 3 — Supplementary Material 3 [file 41598_2025_21285_MOESM3_ESM.png]

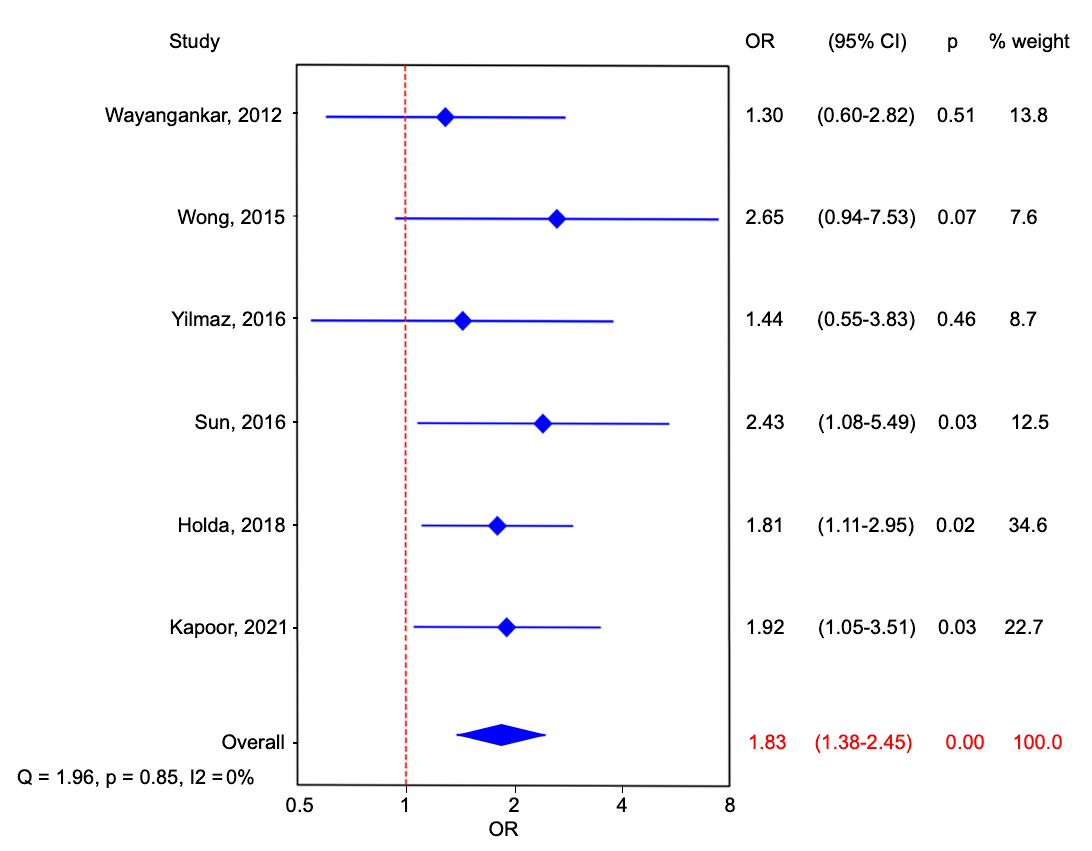

Supplement: Supplementary file 4 — Supplementary Material 4 [file 41598_2025_21285_MOESM4_ESM.png]

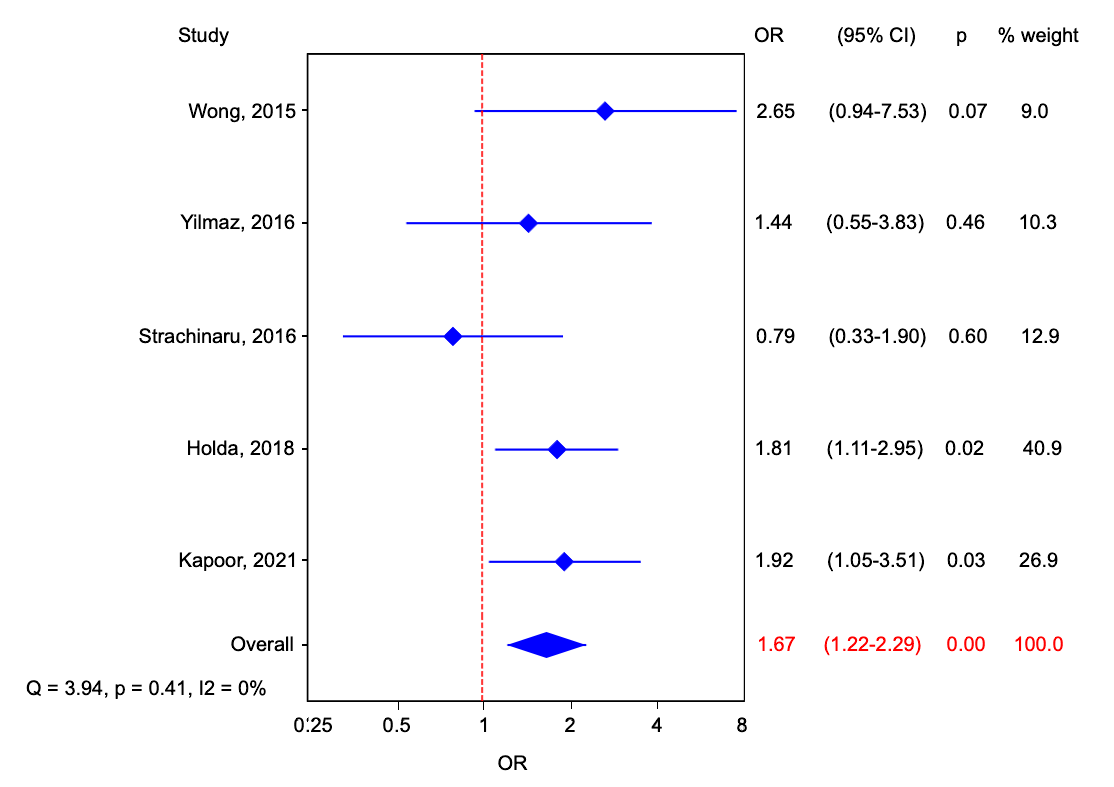

Supplement: Supplementary file 5 — Supplementary Material 5 [file 41598_2025_21285_MOESM5_ESM.png]

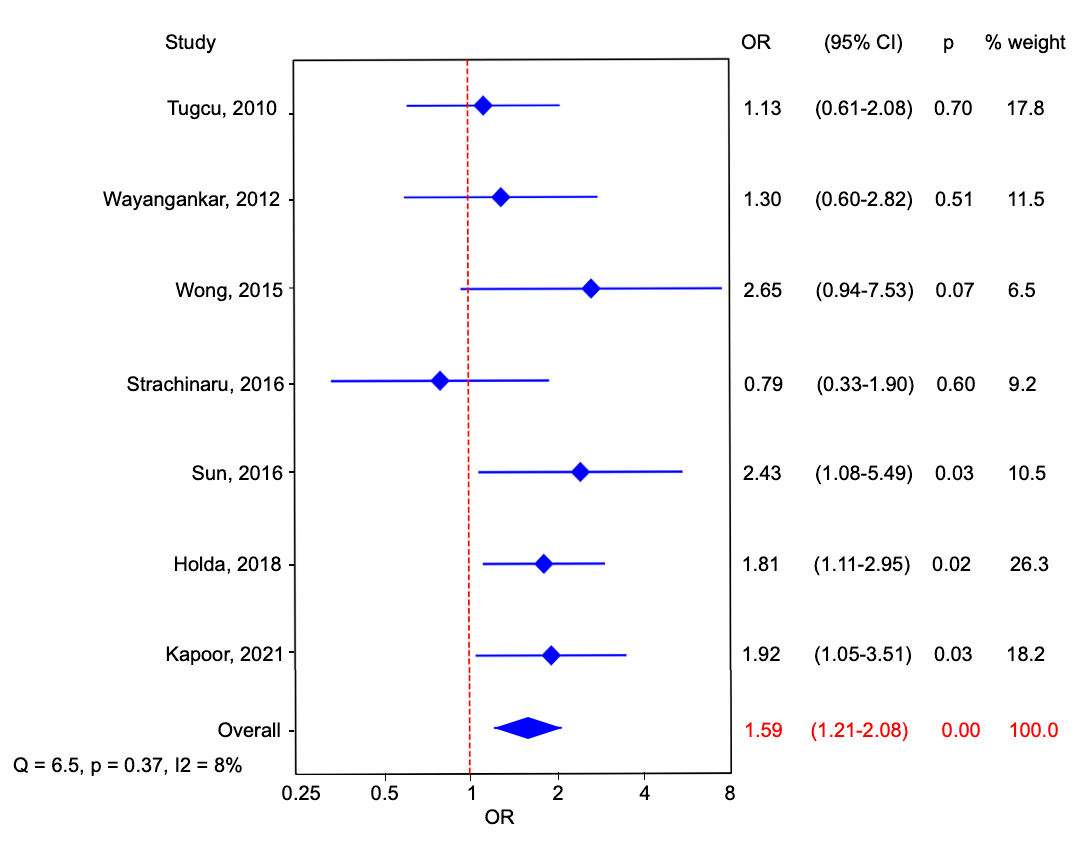

Supplement: Supplementary file 6 — Supplementary Material 6 [file 41598_2025_21285_MOESM6_ESM.png]

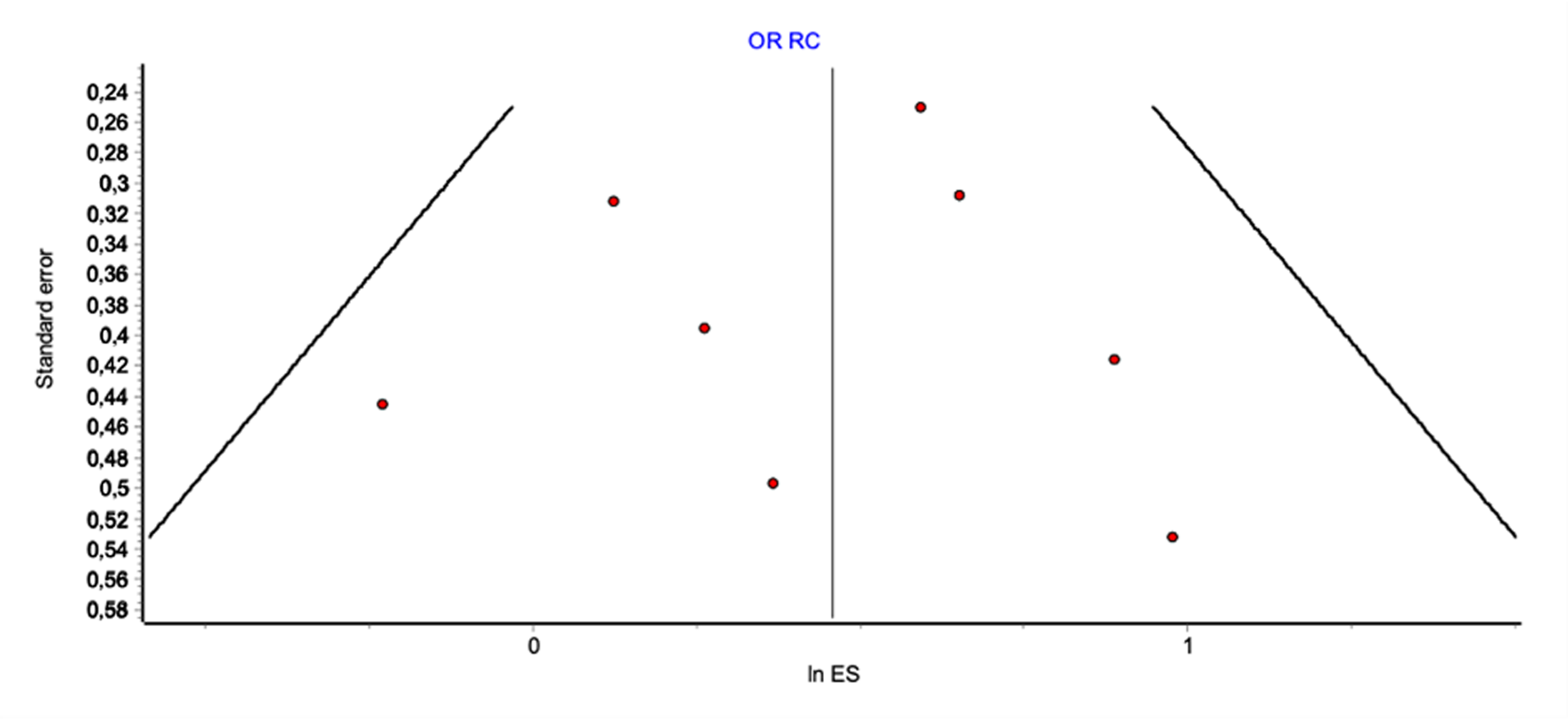

Supplement: Supplementary file 7 — Supplementary Material 7 [file 41598_2025_21285_MOESM7_ESM.png]
